# Recent Trends and Practices Toward Assessment and Rehabilitation of Neurodegenerative Disorders: Insights From Human Gait

**DOI:** 10.3389/fnins.2022.859298

**Published:** 2022-04-15

**Authors:** Ratan Das, Sudip Paul, Gajendra Kumar Mourya, Neelesh Kumar, Masaraf Hussain

**Affiliations:** ^1^Department of Biomedical Engineering, North-Eastern Hill University, Shillong, India; ^2^Biomedical Applications Unit, Central Scientific Instruments Organisation, Chandigarh, India; ^3^Department of Neurology, North Eastern Indira Gandhi Regional Institute of Health and Medical Sciences, Shillong, India

**Keywords:** gait, inertial sensor, myography, plantar pressure, postural balance, wearable sensors, neurological disorder, phytochemical

## Abstract

The study of human movement and biomechanics forms an integral part of various clinical assessments and provides valuable information toward diagnosing neurodegenerative disorders where the motor symptoms predominate. Conventional gait and postural balance analysis techniques like force platforms, motion cameras, etc., are complex, expensive equipment requiring specialist operators, thereby posing a significant challenge toward translation to the clinics. The current manuscript presents an overview and relevant literature summarizing the umbrella of factors associated with neurodegenerative disorder management: from the pathogenesis and motor symptoms of commonly occurring disorders to current alternate practices toward its quantification and mitigation. This article reviews recent advances in technologies and methodologies for managing important neurodegenerative gait and balance disorders, emphasizing assessment and rehabilitation/assistance. The review predominantly focuses on the application of inertial sensors toward various facets of gait analysis, including event detection, spatiotemporal gait parameter measurement, estimation of joint kinematics, and postural balance analysis. In addition, the use of other sensing principles such as foot-force interaction measurement, electromyography techniques, electrogoniometers, force-myography, ultrasonic, piezoelectric, and microphone sensors has also been explored. The review also examined the commercially available wearable gait analysis systems. Additionally, a summary of recent progress in therapeutic approaches, viz., wearables, virtual reality (VR), and phytochemical compounds, has also been presented, explicitly targeting the neuro-motor and functional impairments associated with these disorders. Efforts toward therapeutic and functional rehabilitation through VR, wearables, and different phytochemical compounds are presented using recent examples of research across the commonly occurring neurodegenerative conditions [viz., Parkinson’s disease (PD), Alzheimer’s disease (AD), multiple sclerosis, Huntington’s disease (HD), and amyotrophic lateral sclerosis (ALS)]. Studies exploring the potential role of Phyto compounds in mitigating commonly associated neurodegenerative pathologies such as mitochondrial dysfunction, α-synuclein accumulation, imbalance of free radicals, etc., are also discussed in breadth. Parameters such as joint angles, plantar pressure, and muscle force can be measured using portable and wearable sensors like accelerometers, gyroscopes, footswitches, force sensors, etc. Kinetic foot insoles and inertial measurement tools are widely explored for studying kinematic and kinetic parameters associated with gait. With advanced correlation algorithms and extensive RCTs, such measurement techniques can be an effective clinical and home-based monitoring and rehabilitation tool for neuro-impaired gait. As evident from the present literature, although the vast majority of works reported are not clinically and extensively validated to derive a firm conclusion about the effectiveness of such techniques, wearable sensors present a promising impact toward dealing with neurodegenerative motor disorders.

## Introduction

Human gait refers to the way an individual walks. It is a cyclical process with various phases, and each step contributes to one of the significant tasks responsible for locomotion, viz., weight acceptance, balance, and limb advancement. Human gait has been widely studied in healthy individuals and various pathologies to understand the mechanisms of movement and balance disorders. Alteration of synchronous coordination of multiple muscles and the neuro-motor system can cause atypical gait generation. Factors like accidents, aging, and neurological impairments cause degeneration of the musculoskeletal system, resulting in gait abnormalities. In turn, a pathological gait can significantly reduce the quality of life in terms of mobility and other psychological factors. One of the leading causes for gait impairment is neurodegenerative disorders like Parkinson’s disease (PD), multiple sclerosis (MS), Alzheimer’s disease (AD), Huntington’s disease (HD), Amyotrophic Lateral Sclerosis (ALS), along with certain forms of dementia. Although, as per the prediction of the World Health Organization (2006), neurological disorders were to contribute as the second leading cause of worldwide deaths, this figure was surpassed almost one and half decades ahead of its predicted time frame (Feigin et al., 2017, 2019). PD is one of the major neurodegenerative disorders of the central nervous system (CNS), affecting motor and non-motor functions, including gait and posture (Emamzadeh and Surguchov, 2018). Hip and knee are the two major contributors to non-neurological gait abnormalities (Pirker and Katzenschlager, 2017).

**FIGURE 1:**
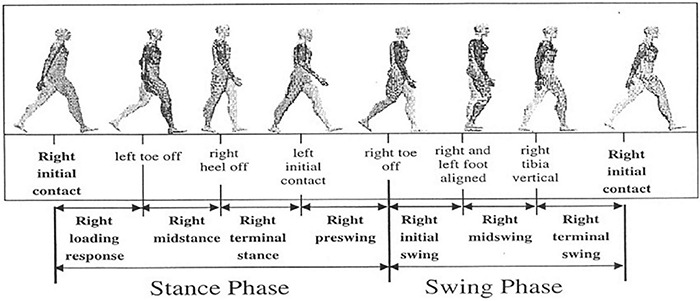
Gait phases and used terminologies to partition the phases. For the representational purpose, the gait segmentation of the right leg (ipsilateral) is described w.r.t. the left (contralateral) leg. [Image source: Li et al. (2016a), the open-access article under the CC BY-NC-ND license].

Gait events like heel strike (HS) of the foot [often denoted as Initial Contact (IC)] as well as the toe off (TO) signify the phase shift between stance and swing phase. Although these events are general indicators of typical gait phases and appear sequentially in gait timelines, these might be missing in some pathological cases. Figure 1 shows the conventional events associated with walking and different phases of the human gait cycle. Previous literature has reported gait kinetics and kinematics, spatiotemporal, mobility, balance, rhythm, etc., as potential inputs for classifying gait patterns of healthy controls from PD (Wahid et al., 2015; Papavasileiou et al., 2017; Suppa et al., 2017), HD (Kegelmeyer et al., 2017; Purcell et al., 2019), Hemiplegia (Lemoyne and Mastroianni, 2020, 2021), and stroke (Mannini et al., 2016; Papavasileiou et al., 2017). Gait parameters are a practical input in monitoring and quantifying therapeutic progress (Badaru et al., 2012). An in-depth study of any individual’s gait can give information varying from kinetic and kinematic aspects to different musculoskeletal functions (Li et al., 2016a). Hence, gait analysis finds application in clinical diagnosis, rehabilitation, sports, and biometric security. This article presents a review of the latest advances in wearable sensors and techniques for ambulatory gait analysis, focusing on clinical aspects of neurodegenerative gait disorders. The role of different sensors in studying various facets of clinical gait analysis, including event detection, spatiotemporal parameter measurement, joint kinematics analysis, gait investigation, and postural balance analysis, have been methodically reported. A total of 48 original research articles published from 2005 to 2021 have been thoroughly discussed, along with several other technical and review papers. In addition, four commercially available products and their application have also been outlined. Section 2 presents an overview of the most prevalent neurodegenerative gait disorders and their neuromuscular motor implications. Section “Gait Analysis: Terminologies and Techniques” gives an overview of various practiced modalities of a conventional clinical gait analysis over the years and how the advancement of wearable technology has led to the shift from stationary ambient sensor-based measurement setups to body-mounted techniques. Section “Wearable Sensors for Gait Parameter Estimation” offers a detailed review of the sensing techniques for general and neurodegenerative gait disorders. This section predominately highlights the role and application of inertial sensors in almost all spectrums of gait analysis. Other methods such as force sensor-based insole and electromyography are also discussed in detail. Apart from these conventional methods, different miscellaneous sensors that have been attempted or show promising performances are also highlighted. In addition, the review also discusses some commercially available measurement systems. Section “Advances in Therapeutic Intervention” introduces the recent progress toward a non-pharmacological intervention to mitigate the challenges of neurodegenerative motor and functional impairments. Section “Discussion and Future Direction” includes a discussion and the authors’ comments about the reported techniques and the future trends. Each unit is presented with a short concluding remark by authors emphasizing relevant context. The review concludes with a summary of the article.

## Neurodegenerative Gait Disorders

Neurodegenerative diseases are a heterogeneous group of progressive disorders associated with degeneration of the central or peripheral nervous system. They alter the nervous system’s structural, biochemical, electrical, and functional activities. This results in a loss of coordination of the neuromuscular system, causing gait and balance disorders (Cicirelli et al., 2021).

### Alzheimer’s Disease

Alzheimer’s disease is the most prevalent neurodegenerative disorder that affects the patient’s memory and comprehension early and eventually leads to neuromotor impairments. Although the exact pathology behind AD isn’t ascertained, studies have reported mitochondrial dysfunction (Correia et al., 2012; Carvalho et al., 2015; Rahman and Rhim, 2017) and alteration of axonal transport (AT) (Wang Z. X. et al., 2015) as contributing agents for neurodegenerative diseases like AD. In the late stages, the most common motor impairments include bradykinesia (slow movement), extrapyramidal rigidity, and gait disorders. Such patients exhibit reduced gait speed and step length, decreased gait frequency (cadence) and increased variability (Pieruccini-Faria et al., 2021). A recent study (Tian et al., 2021) has shown that older persons with slow gait speed and less fragmented activity are at higher risk of developing AD, thereby presenting gait as a potential early indicator for predicting the disease.

### Parkinson’s Disease

Parkinson’s disease is the second most common neurodegenerative disease, resulting from neuronal cell loss in the mid-brain substantia nigra pars compacta region and dopamine (DA) depletion in the striatum (ST) (Bastide et al., 2015) with multiple neurotransmitters deficits (Marinus et al., 2018). In addition, AD associated pathology like mitochondrial dysfunction is also commonly present (Carvalho et al., 2015; Rahman and Rhim, 2017). Accumulation of misfolded α-synuclein, often related to different neurotoxin pathways, is another distinct hallmark of PD (Chen et al., 2019) and can cause neuroinflammation, oxidative stress, and induced endoplasmic reticulum (ER) stress in such patients. This neuro-disorder affects up to 10 million people worldwide (Emamzadeh and Surguchov, 2018). Oxidative stress caused due to imbalance of free radicals’ homeostasis in the body causes cellular and tissue damage. PD involves the primary type of hypokinetic movement disorder resulting in bradykinesia, hypertonia (rigidity), tremor, and flexed posture, marked by reduced gait speed and step length, festination (Vallabhajosula et al., 2013). A typical condition during severe stages of the disease is the freezing of gait (FOG). It is defined as a brief, episodic absence or marked reduction of forwarding progression of the feet while walking, turning, or initiating gait, despite the intention to walk. FOG episodes are one of the primary reasons for losing balance and increased risk of falls in such patients.

### Huntington’s Disease

The Huntingtin (HTT) gene mutation, due to expansion of CAG triplet (cytosine, adenine, and guanine), leads to polyglutamine tract elongation is often linked to HD (Xu et al., 2017). In addition, the formation of Reactive Oxygen Species (ROS) due to oxidative stress is also considered a significant trigger for it (Manoharan et al., 2016). Increased ROS generation is also related to the accumulation of proteins such as α-synuclein in PD (Follett et al., 2016). HD is clinically characterized by involuntary movements such as chorea, psychiatric signs, and progressive dementia. Such patients struggle with uncontrolled movements and loss of cognitive abilities. With disease progression, poorly coordinated body movements and unsteady gait become more visible. Their gait is characterized by slow speed, reduced stride length, variable stepping pattern, and increased stance-to-swing duration ratio. All these factors, in turn, increase the risk of falls in HD patients, thereby further limiting their functional capacity (Georgiou et al., 2020).

### Amyotrophic Lateral Sclerosis

Amyotrophic lateral sclerosis is pathogenically characterized by enormous oxidative stress and mitochondrial dysfunction. It affects the motor neurons of the cerebral cortex, brain stem, and spinal cord. This disruption of communication between the cerebrum and muscle results in muscle atrophy improper limb functioning, resulting in altered gait patterns. Gait dysfunction in ALS is distinguished by increased inter-stride fluctuation, small duration on a single limb, small step length, and decreased cadence (Garcia-Gancedo et al., 2019).

### Multiple Sclerosis

Multiple sclerosis is a demyelinating, chronic, and progressive disease-causing episodic deterioration of neuromuscular functions caused due to autoimmune-mediated loss of myelin and axonal damage (Jones et al., 2017). Clinical symptoms of MS include sensory, cognitive, and motor impairment. With progression over time, the gait shows distinguishable signs of decreased gait speed, step length, cadence, and joint range of motion (ROM) with increased inter-stride gait variability. Such patients also exhibit poor postural control (Kalron et al., 2016).

To summarize, the literature shows that gait and functional impairments are prevalent in subjects with neurodegenerative disorders. So, gait and balance analysis as a clinical tool is helpful for better diagnosing and managing such impairments. In many cases, locomotor impairments are the earliest manifestation of the disease, such as PD. Also, different neurological disorders often appear with overlapping clinical symptoms, such as dementia. However, assessment of movement and balance-related parameters can help diagnose the exact underlying cause. Thus, gait and postural analysis are increasingly used in clinical setups for diagnosis, progression monitoring, and providing targeted therapy.

## Gait Analysis: Terminologies and Techniques

Gait analysis refers to the systematic measurement, description, and assessment of parameters significant to human motion. These parameters can vary from ground reaction force (GRF) and joint torque to joint kinematics like range of motion (ROM) and segment acceleration. A systematic gait analysis includes structuring information, observation of a strict pre-assigned protocol, and a method for data interpretation. General gait analysis is performed from the perspectives of extracting kinematic, kinetic, or Spatiotemporal gait parameters. During the clinical gait study, two effective practices were followed: semi-subjective analysis and objective analysis. Specialists and experts usually perform semi-subjective measurements based on a predefined set of observations and questionnaires. The subjects must perform certain activities in a pre-determined *walking circuit* with clear markings, and the clinician records the parameters of interest with simple tools like a stopwatch. Although such methods are practical in settings with no access to sophisticated gait measurement systems, such measurements are primarily inaccurate, require expertise, and are highly subjective processes. It is almost impossible to observe/record multiple contrasting parameters simultaneously. On the other hand, the objective-based analysis uses sophisticated devices and equipment to measure a wide range of parameters simultaneously. These devices can be based on image processing techniques, floor-mounted measurement systems, or body-mounted instruments.

Objective assessments based on quantitative gait measurements are desired for practical gait analysis and rehabilitation planning. However, 3D gait analysis, which is conventionally conducted using motion capture cameras and force platforms, requires technical expertise and is inaccessible to most clinics and hospitals in rural settings due to high cost. Gait data outputs can include many variables, which may be overwhelming for clinicians to interpret, adding to the challenges of using gait analysis in clinical settings. Therefore, a low-cost, portable, user-friendly instrumented method that quantifies gait has broad clinical application for monitoring individuals’ gait parameters and physical activities in outpatient clinics, community, and home settings. An objective, automated process of quantifying and assessing gait pathology can allow clinicians to invest their resources and time in prescribing effective and more targeted treatments. User-friendly systems for measuring and tracking gait can also aid in assessing the quality of life.

The earliest modern studies on human walking kinematic were performed by Marey and Muybridge using still cameras in the 1870s (Whittle, 1996). With the use of television cameras during the seventies, the process became quicker and more convenient since these cameras were linked directly into computers (Whittle, 1982). Several research groups developed a kinematic gait measurement system using this approach, and several of these early systems evolved into commercial equipment.

Gait kinetic measurement-based studies have mainly dealt with the forces generating during the ground-foot interactions, primarily measured using force platforms. The force platforms have evolved from purely mechanical one-dimensional design (Amar, 1920) to more accurate 3D instruments with digital outputs. Although, until recently, most research on gait kinetic analysis was focused on ground reaction forces, modern gait analysis systems also provide a measure of joint moments and joint powers.

Based on the requirement and technique of measurement, a clinical gait analysis is targeted either at gait event detection and segmentation, spatiotemporal parameter estimation, or gait classification. A wide range of sensors and systems are developed and used for capturing these signals. These devices and techniques used for gait investigation can be classified based on the placement and positioning of the measurement system: Wearable sensors (WS) and Non-wearable sensors (NWS). A WS system is mandatorily placed on different body segments like the foot, shank, pelvis, etc. For example, data from an inertial sensor (accelerometer and gyroscope) set around the ankle joint can be used for distinguishing a healthy gait from a person with Friedrich’s ataxia (Lemoyne et al., 2016). State-of-the-art NWS systems, colloquially also referred to as ambient sensors, consist of two distinct technological approaches: (a) optical motion capture systems that track targeted joints and orientations while walking, either in 2D or 3D and (b) floor sensor-based plantar profile measurement systems. Again, the optical motion capture systems have two families: reflective markers (Roy et al., 2020; Wang et al., 2021) and markerless camera systems (Zago et al., 2020; Hazra et al., 2021). The marker-based systems compute the position of joints and the orientation body segments through the 3D localization of the body markers using multi-camera stereophotogrammetric video systems. A markerless camera system uses the human body model and image features to determine the shape, pose, and joint orientations. The floor sensor-based measurement method relies on pressure sensing technology. Force plates, consisting of load cells, measure the 3D GRF and moments involved during human locomotion. On the other hand, a pressure platform records the plantar pressure profile variation during gait, revealing critical information regarding foot loading pattern and CoP progression. Such a platform consists of arrays of capacitive/resistive sensitive cells that measure pressure acting on each cell due to foot-ground interaction. Both force plate and pressure platform are usually floor mounted, and thus several gait steps can be recorded from them depending on the size of the same. However, a class of treadmill-mounted force/pressure measurement systems allows a larger volume of gait data recording (Wiik, 2016). All NWS systems are operated in controlled facilities, and subjects must follow a predefined protocol. Some measurement methods involve a combination (hybridization) and are essentially a laboratory confined practice. However, the current gold standards for gait analysis are NWS-based measurement tools and consist of either a force plate, an instrumented walkway, or a motion capture system. These measurement methods provide excellent quality data with high accuracy and repeatability. However, high-cost setups provide limited capture volume and a specialized workforce to operate the instruments. Work on compact and ambulatory gait sensors (WS) has picked up the pace at different research groups to overcome these limitations. Such a measurement system provides an alternative to conventional laboratory-based measurements at the cost of reduced accuracy and reliability. Table 1 highlights some of the characteristics associated with both techniques.

**TABLE 1:** Comparison of NWS and WS measurement systems.

NWS	• Accurate, precise, and repeatable measurements
	• Free from environmental interference
	• Multidimensional feature sets can be extracted
	• No restriction of power consumption
	• The number of gait cycles that can be recorded depends on the dimension of equipment and room
	• High cost and bulky equipment confined to laboratory space
	• Requires comparatively higher subject preparation time and stringent protocols; often leads to biased walk from the subject
	• Not suitable for outdoor applications and continuous data monitoring
WS	• The portable, low-cost, miniaturized system that can be easily integrated into electronic systems
	• No need for a controlled environment; the application can be extended to indoor as well as real-life scenarios
	• It can be used for feedback in real-time control applications like orthosis/prosthesis control
	• The range of extracted gait features generally is low. However, with intelligent and powerful computing techniques, new features can be added
	• Requires complex data processing tools to tackle noise and external interferences
	• Sensor placement location and attachment is a significant issue
	• Restriction of power consumption

The distinct advantages possessed by WS in terms of proving almost natural agility over long-term measurements have made them a popular choice for use in ambulatory gait analysis. Such a method allows researchers and clinicians to record physiological features constantly. Therefore, the demand for such technology, which measures gait characteristics either for activity recognition or gait event classification, has risen significantly in the last two decades.

Literature shows that gait analysis is a multidimensional approach depending on the parameter of interest. The gait and balance features can vary from kinematic kinetic to physiological aspects, presenting a vast gait feature set. However, a current technique to extract these features is broadly grouped into two classes, based on the position of the measurement device. The ambient or non-wearable sensors, although being gold standards for gait measurements, possesses specific challenges, thereby limiting their clinical translation. On the other hand, wearable devices show promising performance for future clinical gait analysis. Therefore, this review presents an overview of advancements in wearable technologies aiming to mitigate the challenges associated with ambient sensors.

## Wearable Sensors for Gait Parameter Estimation

Wearable gait analysis tools generally comprise different sensing principles, mainly inertial, force, flexible goniometers, and myoelectric. Few additional sensors, such as electromagnetic tracking systems, sensing fabric, ultrasonic sensors, etc., have also been reported. A single type or combined multiple sensor system (multimodal) may be used based on the target application. However, the wearable gait analysis-based technique is still in its infancy. There is no consensus regarding a set of derived gait characteristics to be assessed and their clinical relevance (Beauchet et al., 2017). The basic principles, features, and overview of reported works around these sensors and systems are described in this section.

### Inertial Sensors

Inertial sensors (IS) or Inertial Measurement Units (IMU), comprising accelerometers and gyroscopes and sometimes magnetometers, measure an object’s motion dynamics, viz., orientation, velocity, acceleration, and gravitational forces. An accelerometer measures acceleration along its sensitive axis and effectively measures the gait motion. The acceleration/velocity of the feet or legs in the gait has been determined to perform the gait analysis (Zeng and Zhao, 2011; Hsu et al., 2014). A gyroscope measures the angular rate and can be incorporated to measure the motion and body segment orientation (Catalfamo et al., 2010; Ayrulu-Erdem and Barshan, 2011). The commercial availability of MEMS-based IS, small form factor, and accessible electronic integration is the critical factor that truly opened up new perspectives in human movement analysis, which justifies the significant literature-related exploitation during the last two decades (Tao et al., 2012; Muro-De-La-Herran et al., 2014; Caldas et al., 2017; Bowman et al., 2021).

**FIGURE 2:**
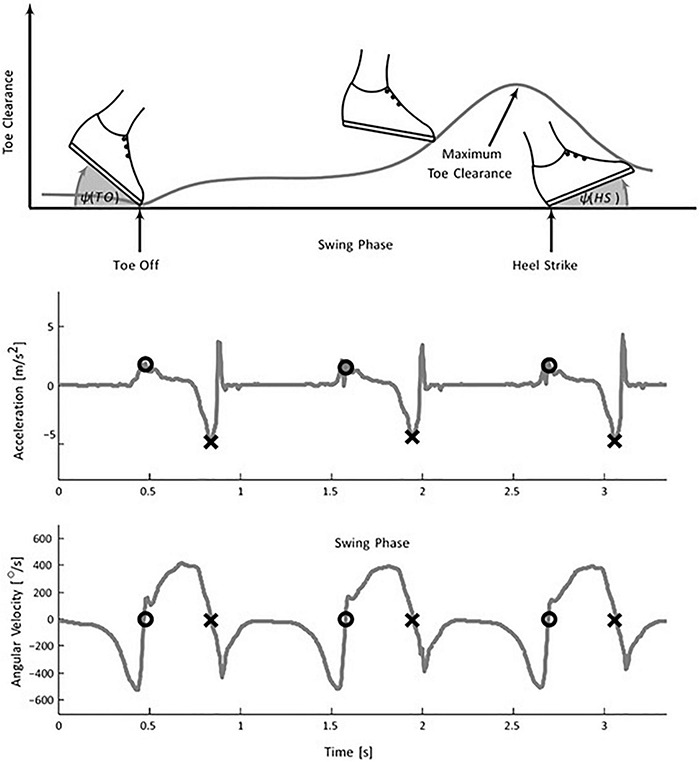
Foot mounted inertial gait pattern in the sagittal plane. (Top) the plot shows a variation of foot angle (w.r.t. ground) at TO, HS, and toes clearance during the swing phase. The negative peak of the acceleration signal determines the HS (middle plot) whereas (lower plot) TO is extracted using the zero crossing of the angular velocity signal [Image source: Schülein et al. (2017), under Creative Commons Attribution 4.0 International License].

Morris (1973) marked the beginning of wearable sensor-based ambulatory gait analysis using accelerometers. The author used six uniaxial accelerometers mounted on a rigid bar for solving the equation governing the motion of a rotating rigid body and determining its angular acceleration. IS-based gait analysis since then has been used for a wide range of measurements: from event detection, kinematic, and kinetic parameter estimation to gait classification. Inertial sensors, placed at different limb segments such as foot, shank, hip, etc., produce a repeatable pattern of signals that signify specific gait events. Many algorithms and methods like thresholding (Ledoux, 2018), peak detection (Lee and Park, 2011; Maqbool et al., 2016; O’brien et al., 2019), zero-crossing (Hundza et al., 2013; Formento et al., 2014; Gouwanda et al., 2016; Maqbool et al., 2016; O’brien et al., 2019), angular rate reversal (Hundza et al., 2013; Schülein et al., 2017), Linear Discriminant Analysis (LDA) (Jiang et al., 2018) have been proposed and developed to identify gait events like HS, TO, and flat foot (FF) on real-time as well as offline mode. For example, the TO and HS detection work using a single gyroscope placed at shank by Hundza et al. (2013) uses rate reversals and zero crossings of the angular rate pattern. Figure 2 shows typical acceleration and angular rate trajectories of foot-mounted IMUs (Schülein et al., 2017). The algorithm satisfactorily identifies events with 100% accuracy in the case of healthy controls and PD patients. However, the method needs manual adjustment of parameters to process data with varied angular rates to reject any false peaks.

Rampp et al. (2015) presented a method for detecting gait events and subsequently computing clinically relevant temporal and spatial parameters like stride length, stride time, swing duration, and stance time using an accelerometer and gyroscope. The stride length was calculated by double integrating the acceleration data obtained from an accelerometer. A work on TO detection for level ground and transition on-ramps was reported by Joshi et al. (2016) using a tri-axial accelerometer placed at the dorsum of the foot. The method uses the wavelet decomposition technique of foot acceleration data to derive a unique feature in a particular frequency band, yielding estimated TO occurrence. For repeatable reference of TO, foot switches were placed below the foot. The work also reported the detection of a transition from a level ground walk to ramps. However, as noted, the algorithm is limited to the identification of TO alone and has not been validated for detecting HS. Das et al. (2019) proposed a method based on the variation of foot inclination angle, measured using an inertial sensor-based wireless foot sensor module (WFSM) placed at the dorsum of the foot during walking to identify HS TO. The detection algorithm uses a peak detection and heuristic approach to mark these events.

The choice of the detection algorithm is based on the position of the sensor attachment as the signal patterns are strictly a function of the sensor placement on limb segments. The methods discussed above have the advantage of being easy to implement and require less computation power and tools. However, inherent noise in raw data, like multiple peaks and thresholds from inertial sensors, yields poor detection when gait is altered (Cuesta-Vargas et al., 2010; Chalmers et al., 2014). Techniques such as Fuzzy Logic (Alaqtash et al., 2011), Machine Learning (Mannini et al., 2016; Hannink et al., 2017), Neural Networks (Sun et al., 2012; Dehzangi et al., 2017) have also been used to overcome this. Such practices show improved accuracy and can handle complex signals. However, they require high computation time and power and thus are not always apt for real-time applications.

**FIGURE 3:**
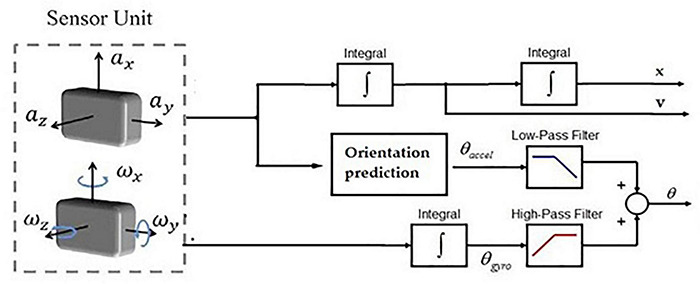
Schematic for IMU-based gait kinematics measurement.

Although the earliest application of inertial sensors, especially accelerometers, was for gait event detection, most recent reports targeted kinematic gait features (Seel et al., 2014; Picerno, 2017; Dorschky et al., 2019). The first study that formalized estimating joint kinematics using inertial sensors (accelerometers) dates to 1990 (Willemsen et al., 1990). Accelerometers have been widely used for static tilt calculation, while a gyroscope has frequently been used for estimating rotational angles. A fusion of both these sensor inputs can yield information regarding joint angles and positions. Figure 3 shows the typical functional block for inertial-based estimation of parameters such as linear acceleration, position, tilt angle, etc.

Measurement of position and range of motion (ROM) of lower limb joints, viz., knee (Willemsen et al., 1990; Tong and Granat, 1999; Dejnabadi et al., 2005; Cooper et al., 2009; Favre et al., 2009; Takeda et al., 2009; Djurić-Jovičić et al., 2011; Seel et al., 2014), hip (Cutti et al., 2010; Horenstein et al., 2019; Teufl et al., 2019b), ankle (Kwakkel et al., 2007; O’donovan et al., 2007; Chang et al., 2016), and entire lower limbs (Findlow et al., 2008; Picerno et al., 2008; Sun et al., 2016), is one of the leading reported methods toward gait kinematics. Such methods rely on either calculation of individual segment kinematics (mainly in the hip and ankle) or the relative orientation between the proximal and distal sensor-embedded frames (in the case of knee joints).

Dejnabadi et al. (2005) presented a novel method for measuring the absolute angle of knee rotation by estimating the joint center of rotation acceleration. Two accelerometers were placed at the shank and thigh region, and their projections (virtual sensors) were set to adjacent segments at the center of rotation. As a result, the absolute value of knee joint rotation could be measured rather than the relative angle (commonly measured using information about adjacent limb inclination). The method thus eliminates the need for integration or cyclical nature to compute the angle, thereby minimizing integration or drift errors. However, subject-specific modeling with prior anatomical information is required.

A recent application of IMUs in movement analysis is toward gait and postural balance study, especially for patients with balance disorders like AD, PD, mild cognitive impairment (MCI), etc. Hsu et al. (2014) proposed an automated algorithm with input from a hip-mounted accelerometer for studying gait balance in AD and healthy controls (HC). The acceleration data of the waist was used to compute the signal vector magnitude and subsequently the 3-axis directional angle. The projection displacement of each step, estimated using the inclination angle, is translated in postural sway in the anterior-posterior and medial-lateral directions. The method was validated on a cohort of 21 AD and 50 HC under eight standard balance test protocols like Eyes open/close, single foot tandem stand, etc.

Wang Z. X. et al. (2015) presented an automated algorithm for performing the *time-up and go test* (TUGT) using inputs from 3 IMUs, one each placed at hip and both feet. A unique motion signature pattern, based on a pre-set threshold, recorded from the coronal plane is segregated to identify the start point and endpoint of the stand-up period and sit-down. The time required by an individual to perform, sit, stand, and finally complete the TUGT is calculated based on these events. A study with 46 subjects (21 AD + 25 HC) was performed to validate the proposed methodology.

One of the most detailed works on balance analysis using IS-based measurement is reported by O’brien et al. (2019) using a single IS placed at the hip. Gait events, namely HS and TO, were detected by finding the local minima and local maxima of the continuous wavelet transformed vertical acceleration component. The angular velocity along the vertical axis filtered through a fourth-order low pass Butterworth filter identifies the left and right foot. The lower limbs were modeled using an inverse pendulum model (IPM) for spatial feature estimation. The change in the height of CoM (obtained by double integration of vertical component of acceleration) was scaled in terms of step length. The frequency-domain features were extracted using the Bergs Balance Scale (BBS) for the static postural balance study. In contrast, the time-domain features were evaluated from acceleration and its differential and integral outputs. Table 2 tabulates significant inertial sensor-based gait analysis targeted at event detection, parameter estimation, and balance analysis.

**TABLE 2:** Inertial sensor-based gait and balance analysis: from the prospect of event detection, spatiotemporal parameter estimation, joint ROM, and balance analysis.

Reference	Parameter(s)	Technique(s) used	Subject(s)	Calibration/validation technique	Remarks
**Event detection/temporal parameter measurement**					
(Jasiewicz et al., 2006)	HS and TO	One gyroscope, two linear accelerometers, peak detection, zero crossing, heuristic	26 HC + 14 SCI + Charcot-Marie-Tooth (CMT)	Foot switches	TO latency 50 ms, 100 ms for HS detection; obtrusive due to semi-wired connectivity
(Raveendranathan et al., 2011)	HS and HO	Single 3-axis accelerometer at alternate/multiple positions; peak detection + HMM	1 HC	None	Adaptive to Sensor placement
(Yang et al., 2012)	HS and asymmetry feature	Single accelerometer placed at the lower back, peak detection	15 HC	N/A	No specific accelerometer; The developed iGAIT tool requires manual intervention to set input pre-sets
(Mariani et al., 2013)	HS, TO, HO, and TS	Foot mounted 3D accelerometer+ gyroscope, pitch velocity, negative peak, zero crossing	10 HC, 12 AO, 11 TAR, and 9 AA	Pedar-X Pressure Insole	−33 ± 14 for angular velocity, 81 ± 15 for acceleration
(Hundza et al., 2013)	Temporal, stride length	4 IMU (gyroscope), Y-angular rate reversal	6 (PD) + 7 (HC)	GAITRite, OMC	100% event detection, SD of 6.6 ms and 11.8 ms in HC and PD
(Joshi et al., 2016)	TO	Three-axis accelerometer, wavelet decomposition	6 HC	Foot switch	The transition between level ground and ramps
(Das et al., 2019)	HS and TO	Six-axis IMU, Foot angle variation, peak detection	34 HC	FSR	Improved detection latency of 16 ms
**Joint kinematics and ROM**					
(Dejnabadi et al., 2005)	Knee angle	One IMU (Two accelerometer + one gyro) placed at shank and thigh; virtual projection of physical sensor into rotation joint	8 HC	OMC	Absolute angle calculation with no drift error; subject-specific modeling requires prior anatomical information
(Dorschky et al., 2019)	Hip, knee, and ankle angle	07 six-axis IMU, musculoskeletal model, trajectory optimization	10 HC (M)	OMC	*P* ≥ 0.93
(Gholami et al., 2020)	Hip, knee, and ankle flexion/extension	01 accelerometer placed at foot + CNN	10 HC (M)	OMC	RMSE <3.4% for intra-subject and <6.5% for inter subject
**Spatiotemporal parameters**					
(Salarian et al., 2012)	HS, TO, SL, and gait velocity	Two gyroscopes placed at the shank, a Double pendulum model with two gyroscopes + Fourier series, and most minor square optimization	10 PD, 18 HC, 36 hip-replacement, and seven orthosis	OMC	Validated on a sizeable patient population with multiple disorders
(Takeda et al., 2014)	Gait phases, SL, and L_*Step*_	One IS on each ankle, shank, and thigh; one on the pelvis. Peak detection for events, drift reduction protocol for spatial parameters	5 HC, 10 m walk-test	OMC	Linear drift modeling does not hold for extended walking
(Rampp et al., 2015)	SL, GCT, T_*swing*_, and T_*stance*_	Inertial sensors, Template Search for events	101 NW, 84 WW	GAITRite^®^	0.93 and 0.95 in NW and 0.80 and 0.95 in WW for SL and GCT, respectively
(Wang and Ji, 2015)	T_*stance*_, T_*swing*_, and SL	Foot mounted IS, peak-peak detection+ adaptive thresholding for event detection; CF+ ZUPT+ double integration for SL	15 HC	Non-standard	1.64 ± 0.839 for SL
(Liu et al., 2016)	SL	3D acceleration and angular rate, Dual-ZUPT	14 steps	Videography	
(Ferrari et al., 2015)	GCT, SL, and stride velocity	Foot mounted IMU, Medial-lateral foot angle peak detection for events; KF+ZUPT for stride length	12 HC, 16 PD	GAITRite^®^	Real-time computation on a smartphone, RMSE SL = 4%
(Hao et al., 2019)	SL	3D Euler angle, acceleration, discrete KF, smoother	9 HC (male adults)	OMC	−0.24 ± 1.1 cm for SL
(O’brien et al., 2019)	T_*stance*_, T_*Sw*_, SL, step velocity, and step count	One IMU at hip; Local minima/maxima + Butterworth filter for events; IPM + Double integration for SL	51 HC	GAITRite^®^	Need for additional optimization constant that is derived from GAITRite^®^ for SL estimation
(Das and Kumar, 2021)	SL	Six-axis IMU at foot dorsum; foot angle for gravity compensation and double integration of foot acceleration	10 HC	Zebris walkway, outdoor marking	Acceleration integrated only for swing duration; compensated with foot length
**Spatiotemporal + Joint kinematics**					
(Teufl et al., 2019a)	12 STP including L_*stp*_, step width, 6 DoF kinematics	7 Xsens IS	24 HC (12 M + 12 F)	OMC	Detection means error ∼1.6%, Step width, and swing width *RMSE* > 30%
(Yeo and Park, 2020)	St_*t*_, SL, cadence, step length; knee and hip ROM	Five triaxial accelerometers, gyroscope, and magnetometer (LEGSys+ wearable device) placed at shank, thigh, and pelvis; self-selected walking at the 7-m walkway	30 HC	OMC	The significant difference in hip ROM; measurement within 95% limit of agreement
**Balance**					
(Hsu et al., 2014)	CoM, postural sway rate	Three-axis accelerometer in waist	21 PD + 50 HC	N/A	Validated on a large group; Only static balance
(Wang W.-H. et al., 2015)	TUGT	Three IMUs (1 at hip + 1 at each foot); Signature matching of lateral angular rate + thresholding	21 AD + 25 HC	N/A	Test specific
(O’brien et al., 2019)	10 MWT, BBS, and TUGT	One IMU at hip; FFT+ integration for static balance; Daubechies wavelet approximation for dynamic balance	51 HC	GAITRite^®^	178 features extracted for three balance assessment tests
(Noamani et al., 2020)	Two minutes standing test, inter-segmental moments, and CoP	Accelerometer + gyroscope placed at foot, leg, pelvis, and head-arms-trunk; Musculoskeletal inverse dynamics model	10 HC	OMC+ force plates	Accelerometers alone provide reliable data for standing balance analysis
(Dugan et al., 2021)	Two minutes barefoot standing in EO, EC	17 IMU placed at whole body; jerk index and complexity index from postural sway from pelvis accelerometer	38 concussed patients	N/A	Single accelerometer yields information about postural sway

*HS, heel strike, TO, toe off, HC, healthy control, SCI, spinal cord injury, CMT, Charcot-Marie-Tooth, HMM, Hidden Markov Model, HO, heel off, TS, toe strike, AO, ankle orthosis, TAR, total ankle replacement, AA, ankle arthrodesis, OMC, optical motion camera, CNN, convolution neural network, CF, complementary filter, ZUPT, zero update, KF, Kalman Filter, GCT, Gait cycle time, IPM, inverted pendulum model, St_t_, stance time, T_Sw_, swing time, L_stp_, step length, CoM, center of Mass, TUGT, time-up and go test, 10 MWT, 10 meter walk test, BBS, Berg Balance Scale, AD, Alzheimer’s disease, FFT, Fast Fourier transform.*

To recapitulate, inertial sensors, including accelerometers, gyroscopes, and magnetometers owing to distinct advantages, presents probably the most promising alternative to laboratory-based gait analysis. The measurement from IMU-based methods has been reported for all the domains of gait measurement, i.e., from the very initial step of gait clinical gait assessment (event detection) to its use for automated quantification of disease severity (PD and balance grading). The integration of IMU sensor chips in modern smart devices and their widespread penetration all across society [smartphones (Lemoyne et al., 2010a,b; Mcguire, 2012; Kashihara et al., 2013; Susi et al., 2013; Lemoyne and Mastroianni, 2015, 2018; Sprager and Juric, 2015), wearable bands, etc.] are a positive aspect to look forward to the translation of such technologies for clinical benefit. However, there are still specific challenges to the domain that need to maximize the benefit and make IMU-based devices a conventional gait analysis tool. One of the definite challenges toward fulfilling it is the non-uniformity of technology and use. There is no consensus and guidelines for inertial sensor-based clinical gait evaluation, making it a technology-specific and subjective method. Subject preparation for trials also depends on the specific nature of the targeted parameter, which again is a significant challenge for deployment in clinical settings. Also, from a technology point of use, most of the reported work toward neuro-disorder diagnosis relies on certain aspects and characteristics of the disease. This limits the general use for evaluating other disorders.

### Instrumented Foot Insoles for Gait Kinematics

Floor mounted plantar pressure measurement systems/force plates provide accurate and repeatable measurements. Such systems are widely used for clinical gait assessment and studying gait biomechanics worldwide. However, such stationary platforms have certain demerits, primarily due to the high cost and number of steps that could be recorded. To overcome these limitations, various research groups worked toward developing instrumented force insole that can measure specific plantar kinetic parameters. However, the initially reported methods (Hausdorff et al., 1995; Fraser et al., 2007; Yong et al., 2011) were based on wired communication that often disturbs the natural walk. However, with the advancement of material technology, components of standard plantar force and pressure measurement devices have become readily available in compact and robust forms. As a result, many novel wirelesses-based plantar gait measurement systems have been developed to study gait in naturalistic scenarios over a longer duration (Wahab et al., 2008; Yang et al., 2009; Liu et al., 2010; Crea et al., 2012, 2014; De Rossi et al., 2012; Donati et al., 2013; Howell et al., 2013; Mn, 2014; Qin et al., 2015; Bark et al., 2017; Arafsha et al., 2018; Zhang et al., 2019; Sorrentino et al., 2020; Tahir et al., 2020). While most studies reported the application of commercially available force sensors for their development (Howell, 2012; Howell et al., 2013; Mn, 2014; Qin et al., 2015; Bark et al., 2017; Arafsha et al., 2018; Tahir et al., 2020), few groups designed and fabricated the sensors as well (Wahab et al., 2008; Yang et al., 2009; Zhang et al., 2019; Sorrentino et al., 2020).

One of the detailed and earliest works toward a whole plantar pressure profile measurement system was reported by Howell (2012) and Howell et al. (2013). They reported an instrumented insole with 32 FSRs to cover the entire footprint area with more concentration in pressure hotspots. Each sensor was calibrated for force measurement using a load cell, and the contribution of each calibrated sensor was taken into consideration for measuring vGRF and ankle moment. The loading patterns were studied at each sensor during walking to sort an adequate number of sensors and their best locations. Subsequently, a second prototype was developed with 12 FSRs to measure vGRF, ankle, and knee moments. Using the developed insole and data recorded from the motion analysis laboratory, a database was generated and validated on HC hemiplegic patients using regular ankle-foot orthosis on stroke patients. A linear regression model with inputs from the database was used to develop each subject’s gait models. Testing data for each subject were used to predict the GRF and ankle and knee motion moments with appreciable correlations. Several FSR-based force insoles for gait analysis and related studies have been reported with the interplay of the number and location of sensors, parameters estimated from it, and application area (Liu et al., 2010; Qin et al., 2015; Tahir et al., 2020).

A novel and well-reported system for real-time foot pressure measurement based on silicon cell-based pressure-sensitive pad is reported by the research group of De Rossi et al. (2011, 2012), Crea et al. (2012, 2014), and Donati et al. (2013). The device, consisting of 64 pressure-sensitive elements, is embedded in an insole. The integrated signal conditioning electronic board with wireless communication and power source (battery) is placed inside a casing along the shoe’s lateral side. The authors calculated the vGRF, CoP, and a set of temporal parameters based on the amplitude and activation profile of the sensitive cell and validated against an AMTI force plate. The method thus reported has few distinct advantages in providing high spatial resolution plantar force data, insensitivity to temperature variation, and the need for only calibration. Pressure insoles currently are extensively used for studying gait and balance disorders associated with neurodegenerative motor symptoms, such as detection of FOG episodes in PD (Marcante et al., 2021; Pardoel et al., 2021; Shalin et al., 2021).

### Electromyography

The muscles in a human compose about half of the total body weight. These are composed of bundles of specialized cells capable of contraction and relaxation in response to the stimuli received from the cerebral cortex. The contraction and expansion of skeletal muscles provide the force required to perform various actions in electrical signals. These signals range from a few hertz to 400 Hz and voltages ranging from approximately 10 μV to a few millivolts. The underlying chemical process produces a shortening of the contractile elements of the muscle cell. Electromyography (EMG) is a technique to record and measure muscle activities. Based on the placement of EMG electrodes on the human body, it can be either invasive or non-invasive (also known as surface-EMG).

Surface-EMG (sEMG), in particular, has been exploited for studying muscle activity during dynamic activities such as walking, particularly in gait events and phase recognition for healthy as well as neuro-impaired gait (Ryu and Kim, 2017; Ziegier et al., 2018; Morbidoni et al., 2019; Nazmi et al., 2019; Di Nardo et al., 2020; Keloth et al., 2021; Zahra, 2021; Rezaee et al., 2022). Nazmi et al. (2019) proposed a classification approach for the segregation of stance and swing phase (HS and TO detection) from feeding EMG signal to an artificial neural network. EMG has also been used for intent, like sit to stand detection (Rasool et al., 2012; Chorin et al., 2016; Li B. et al., 2016; Roldán Jiménez et al., 2019; Bhardwaj et al., 2021) and quantitative localized muscle fatigue estimation (Boudarham et al., 2014; Ropars et al., 2016; Roldán Jiménez et al., 2019; Parent et al., 2019) during gait. Although fatigue is considered a multidimensional concept involving both physiological and psychological implications, the former dimension of fatigue can be observed in both the central and peripheral system domains (Zwarts et al., 2008) and is a widely accepted tool for fatigue estimation (Al-Mulla et al., 2011). Analysis of the EMG frequency spectrum (mean, median) is the most widely explored technique for fatigue estimation, and localized muscle fatigue often results in a downward shift of the frequency content of the EMG signals (Naik, 2012). Besides being an everyday activity humans perform in their daily lives, a sit-to-stand task is also commonly used in clinics to evaluate lower limb muscle function. Moreover, it is immensely practiced for therapeutic rehabilitation exercises targeting muscle strengthening, balance improvement, and gait therapy.

Chorin et al. (2016) analyzed EMG and force data from 40 subjects (10 elderly fallers, 30 non-fallers) and concluded that the gastrocnemius lateralis muscle activity differs significantly between fallers and non-faller group. Similarly, Li B. et al. (2016) detected sit-to-stand transitions from the quadriceps EMG data integrated with upper Trunk kinematic data. Roldán Jiménez et al. (2019) evaluated the fatigue during the 30-second sit-to-stand (30-STS) test on a young obese and sedentary woman to ensure fatigue during the trial. The subject was otherwise free from cognitive disorders, musculoskeletal, bone, or joint injury. The muscle activity was measured at six locations of the dominant side (GM, BF, VM, AR, ES, RF, SO, and TA) at a frequency of 1,000 Hz. Muscle fatigue estimation for neurological disorders such as hemiplegia (Boudarham et al., 2013, 2014; Wang et al., 2017; Mazzoli et al., 2018; Fujita et al., 2020, 2021), PD (Huang et al., 2017; Palinkas et al., 2019; Cao et al., 2021), MS (Octavia et al., 2015; Porcaro et al., 2019; Stańczyk et al., 2019) has been reported. The approach for EMG-to-muscle force has also been analyzed in Bogey et al. (2005). Furthermore, EMG signals have been reported to be used to measure other gait characteristics like joint kinematic plots. The joint angular motion recorded simultaneously with EMG data is correlated.

### Miscellaneous Sensors

In addition to the widely used sensing methods discussed above, researchers have explored the possibility of the novel application of alternate sensors/techniques for gait analysis. A few such measurement techniques are discussed below.

#### Force Myography

Force myography measures the external muscle force/pressure generated during human activity. When strapped around a limb circumference using a bracelet/socket, force Sensors measure the outward force developed due to the volumetric changes resulting from the displacement of muscles, tendons, and the skin (Castellini et al., 2014). Research groups have proposed and demonstrated the application of different sensors like piezoelectric (Li et al., 2016b; Ha et al., 2018), capacitive (Truong et al., 2018), flexible fabric (Rasouli et al., 2015), and optical sensors (Fujiwara and Suzuki, 2018). However, the most commonly used sensor for FMG application is FSR, and FlexiForce Sensors, made out of Resistive polymer thick film material (Xiao and Menon, 2019). These methods have been widely used (almost 90% of work-related to FMG) to study and control an upper limb, especially arm-related movements (Xiao and Menon, 2019). Lukowicz et al. (2006) first presented the use of FMG to segregate four types of walking. Signal patterns from FSRs strapped at the thigh were used for classifying locomotion patterns. Yungher et al. (2011) demonstrated the correlation of FMG (surface muscle pressure) with surface muscle electrical activity during gait. FMG signal patterns showed better stride-to-stride consistency than sEMG signals.

Similarly, the study reported in Belyea et al. (2018) also supported the correlation of FMG and sEMG signals, with FMG signals being reported as a better indicator for understanding the fatigue label. Although these studies show potential for application toward gait analysis, the objectives/outcomes of these reported works do not precisely meet the requirement of clinical gait analysis with targeted parameter evaluation. Two recent results (Godiyal et al., 2018; Jiang et al., 2018), using FMG, have been reported for gait event/phase detection.

The work reported by Godiyal et al. (2018) presented a method for detecting HS and TO for overground and ramp walking, including transition. Eight FSRs, evenly placed in a bracelet was, strapped around the subject’s thigh such that each FSR aligned corresponding to prominent thigh muscle. A foot insole system for ground truth/reference data synchronized with the FMG measurement system. A classifier was trained with reference signatures extracted during the training phase that consisted of two signature patterns (one each for HS and TO) for each locomotion mode. Hence, the system could successfully match any of the three-locomotion modes for a test FMG signature. However, the detection framework is subject-dependent as a separate database is created for every trial subject.

**FIGURE 4:**
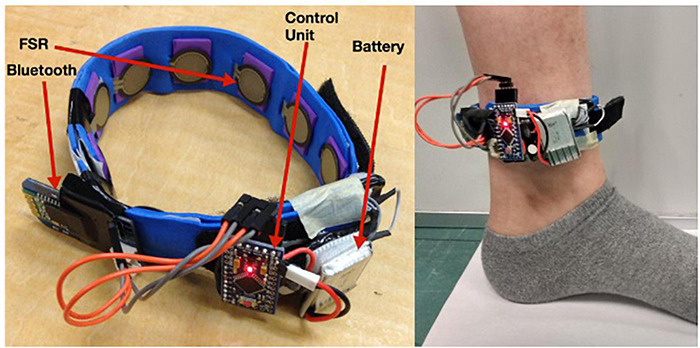
An FSR sensor-based force myography band for gait application [Reproduced with permission from Jiang (2018), Memorial University of Newfoundland].

Another subsequent work on gait phase detection is reported by Jiang et al. (2018) and Jiang (2018) for event detection in treadmill walking. During gait, periodic contraction and relaxation of the extensor and flexor muscles at the ankle alter the pressure distribution. An FMG band, consisting of eight equispaced instrumented FSRs, was strapped around the ankle to record these distinctive FMG patterns (Figure 4). A high-speed motion camera was used for referencing four instances: HS, MSt, PSw, and Sw. A 125 ms sliding window with 93 ms overlap was used to extract a set of 14 distinguishable features, and a Linear Discriminant Analysis (LDA) technique was implemented to classify the gait phases further.

#### Piezoelectric Sensors

Piezoelectric sensors, when stressed, generate electric potential and are often used for dynamic pressure or vibration measurement. The most common piezoelectric sensors are made of either ceramic-based or MEMS-based material. Due to the parasitic effect of piezo materials, the ceramic piezoelectric sensors are not suitable for static or low-frequency measurements (Tahir et al., 2020). However, the MEMS sensors possess distinct advantages: they can be used for 3-axis measurements they generate positive or negative (amplitude) electrical impulses depending on the direction of force acting around each axis. They help detect human motion primarily due to their flexibility, wide frequency range (0.001 Hz to 10 MHz) (Tahir et al., 2020).

Balbinot et al. (2014) designed a footswitch system for HS and TO detection using a piezoelectric transducer out of a buzzer. The Piezo sensors, calibrated for force measurement, were attached beneath a rubber insole at the hallux and heel region. HS and TO were defined as the instances when the force signal exceeded or fell from a threshold of 5N, respectively. These events, thus detected, were further used for deriving a set of temporal gait parameters. The results were validated with a standard force plate (AMTI Inc., United States).

A gait recognition based on flexible piezoelectric sensors [polyvinylidene fluoride (PVDF)] is reported by Cha et al. (2018). Four flexible sensors, one each at the knee and hip of both legs, were attached on an item of loose clothing. The PVDF sensor generates an electric voltage that corresponds to the bending of the corresponding limb joint. The periodic motion component, extracted from the FFT of the time-varying signal, correlates to the gait speed. The system was also able to detect different postural transitions between standing and walking.

Although explored widely, the majority of the piezoelectric sensor-based gait measurement systems focus on analyzing vertical components of pressure/force. However, the shear stress provides vital information especially involving a pathological gait (Hamatani et al., 2016; Jeong et al., 2021). Moreover, crosstalk arising due to the co-existence of piezoelectric coefficients significantly impacts the measurement accuracy (Dai et al., 2020). To address these challenges, recent works (Chen et al., 2020; Dai et al., 2020; Gao et al., 2020) have proposed using multilayer structure with piezoelectric films in distinct polarization orientations (Gao et al., 2020), lamination technique for assembling electrodes with functional films (Dai et al., 2020), and signal decomposition technique to separate the raw signal into several intrinsic mode functions (Chen et al., 2020).

#### Wearable Microphone

The foot is the anatomical structure that is in contact with the ground during walking, and exploration of foot-ground interaction (footstep sound) can reveal important aspects of gait. Microphone sensors, being tiny, low-cost, portable, and readily configurable with embedded electronics, has promising application for capturing footstep sounds generated due to ground-foot impact. Although very limited, footstep sound or gait acoustics have also been explored for gait analysis (Makela et al., 2003; Altaf et al., 2015; Hwang and Gim, 2015). The first wearable acoustic sensor-based gait parameter estimation method was reported by Wang et al. (2016). They presented an event detection method using the wearable microphone to collect footstep sound signals during walking. One microphone attached at the ankle of each foot, such that the microphone holes face toward the ground, was used to collect the sound resulting from different foot parts. Based on the spectral analysis of the sound, a set of 36 features were extracted that included correlation coefficients, energy bands, zero-crossing rate, linear prediction, and mel-frequency coefficients. A support vector machine (SVM) based approach was used to identify and classify step detection, HS, and toe-on, and a set of 9 STP were estimated by fusing both feet sound data. The study was validated on 15 healthy subjects with a detection accuracy of nearly 95%. Jesus et al. (2019) presented a case report on estimation of gait cadence, speed, and stride/step duration for a PD subject (stage 3 on Hoehn and Yahr’s scale) from the gait acoustic signal captured using a microphone. The parameters thus recorded were validated against a motion capture system.

A very recent and exciting feasibility study for using an ear-worn wearable (Earable) microphone for gait identification is proposed by Ferlini et al. (2021). Their work (called EarGate) demonstrates the feasibility of gait identification from walking sound generated and propagated through the musculoskeletal system in the body. The EarGate system employs an inward-facing earphone to leverage the occlusion effect (low-frequency components of a bone-propagated sound when the ear canal orifice is sealed) during the wearer’s movement from inside the ear canal. The proposed method identified 31 healthy subjects with an overwhelming balance accuracy of over 97%. Chiang et al. (2019, 2020) explored the self-doppler effect using a microphone coupled with three buzzers (mounted on a shoe) to detect gait events and compute step length, orientation, and posture.

#### Ultrasonic Sensor

An ultrasonic sensor computes the distance of a target object by emitting short, high-frequency sound waves at regular intervals. When the sound pulse encounters an object in its path, it has reflected the transmitter as an echo signal. This signal reflects a measurement of the distance of the object/target from the source based on flight time. Such time-of-flight-based measurements possess a distinct advantage in suppressing background interference compared to intensity-based measurements. Recent ultrasonic sensors can measure very minute distance change, showing potential for gait analysis application.

Huitema et al. (2002) presented a semi-wearable (hybrid) system for measuring spatiotemporal parameters using ultrasonic sensors. An ultrasonic transmitter, fixed at a stationary position in the room, sends bursts of periodic pulses to the two receivers attached at both shoes of the subject. The propagation delay recorded between transmission and reception is used to localize the position of the subjects’ feet. Information about the dynamic position of the foot and zero foot-velocity phase was used to calculate step length and stride length. Gait events, namely HS and TO, were calculated from the speed plot variation within a single step by applying pre-set thresholds. The study was validated on four healthy subjects.

Another work on the semi-wearable approach by Qi et al. (2014) reported the measurement of 3D foot trajectory using a wireless ultrasonic sensor network. Contrary to Huitema et al. (2002), the ultrasonic transmission unit (mobile) was fixed at the shoe. At the same time, there were four receiving units (anchor), set at 3D planes across the room, all interconnected and synchronized wirelessly. The system records the time-of-flight of the ultrasonic sound waves from mobile to anchors and measures the distance covered by the foot. The 3D foot trajectories thus computed were validated using eight motion camera-based systems while tracking three reflective markers, systematically fixed at the subject’s foot with an RMS error of 4.2%.

### Multimodal In-Shoe Approaches

Researchers all across have been trying to integrate/embed one or more of these techniques, as mentioned earlier, to develop a wearable gait analysis system (especially an in-shoe based) that can deliver the majority of clinically relevant kinetic and kinematic gait parameters. The shoe-based measurement offers distinct advantages in requiring the least preparation time, providing a surface for embedding electronics and hardware with minimal or no aesthetic change. One of the earliest and most comprehensive (and highly referred) works on multimodal shoe-based gait system development is the *GaitShoe* by Bamberg et al. (2008) from the Massachusetts Institute of Technology (MIT). *GaitShoe* includes 3-axis (orthogonal) accelerometers and gyroscopes, four foot-force sensors, two bidirectional bend sensors, two dynamic pressure sensors (PVDF strips), as well as electric field height sensors. The accelerometers estimate SL, SV, and displacement, whereas Gyroscopes measure the foot orientation. The force-sensitive resistors were used for two targets: first to analyze the force distribution under the foot and second to detect gait events, namely HS and TO. The PVDF strips were used as redundant event detection while the electric field sensor analyzed the foot clearance from the floor. The bend sensor records the foot plantar flexion/dorsiflexion variation at metatarsal points. The *GaitShoe* system physically consists of an instrumented insole that houses FSR, PVDF, bend sensor, and field sensor with an attachment housing the accelerometers, gyroscopes, and hardware electronics. The system performance was validated on healthy and pathological (Parkinsonian) gait. However, this system still needs a high level of customization for regular use.

A similar recent system (eSHOE) based on integrated sensors packaged in a shoe is reported by Jagos et al. (2017). The developed wireless eSHOE includes a six-axis IMU with four FSR sensors used for calculating eight temporal parameters: gait cycle time, stance time, swing duration, step time, single support duration, initial double support, double terminal support, and cadence. The system’s performance was validated against GAITRite^®^ walkway, a standard plantar pressure and gait analysis system used widely for computing STP. The measured parameters collected during a study of 10 subjects with a proximal femur fracture from eSHOE showed a significant correlation with the parameter recorded from GAITRite^®^. However, this system functionality is limited to measuring temporal feature sets only, emphasizing spatial features, essential outcomes of clinical gait analysis. Similar in-shoe-based multimodal measurement systems have been reported for application in rehabilitation tasks (Benocci et al., 2009; Edgar et al., 2010; Wada et al., 2010).

### Commercial Ambulatory Wearable Gait Sensors

Research in WS-based gait analysis system development has been ongoing for the last two decades. However, there are minimal commercial WS-based gait analysis systems available in clinics and research setups. One prominent WS system is the Xsens Motion System that uses inertial trackers to capture full-body kinematics of the body with a wireless communication suit. It is widely used in gait motion and biomechanics study (Zhang et al., 2013; Loose and Orlowski, 2015; Schepers et al., 2018) and also serves as a referencing system for validating custom-developed gait measurement methods and protocols (Cutti et al., 2008; Ferrari et al., 2010; Wouda et al., 2016).

Another very recent product based on inertial sensor-based for gait and biomechanics study is “Mobility Lab” from APDM Wearable technologies (Eftekharsadat et al., 2015). The system can perform a standardized test based on clinical protocol, and a patient report is generated for assessment by the clinicians. The system can report performance regarding gait STP, arm kinematics, lumbar postural sway, and sit-stand parameters. Many experimental studies have been reported for studying gait pathologies (Horak et al., 2016; Pal et al., 2016; De Souza Fortaleza et al., 2017; El-Gohary et al., 2017).

An in-shoe plantar pressure measurement system called F-Scan (Júlio et al., 2020) provides dynamic pressure, force, and timing information for foot function and gait analysis. The system targets two user bases: Researchers and Clinicians with additional research software that provides researchers access to raw and processed data in-depth. For medical practitioners, the system generates automated reports. This system is often used to optimize and customize therapy and understand foot biomechanics (Mueller et al., 2003; Thies et al., 2007; Fourchet et al., 2015). Another insole-based plantar force measurement system with integrated IMU is available from Moticon (Moticon ReGo AG, Munich, Germany) (Barratt et al., 2021). It is an entirely wireless device such that the insoles can be embedded into any pair of shoes for continuous and unobtrusive data collection. It also has onboard data recording for outdoor and more extended data recording.

## Advances in Therapeutic Intervention

Despite the widespread prevalence of neurodegenerative disorders, the current treatment (pharmacological) options focus on providing symptomatic relief and slowing down the progression. Presently, no effective drug is available to cure or prevent neurodegenerative disorders. They are also reported to produce adverse health effects. For example, Levodopa (L-dopa) is the most commonly prescribed drug for managing early-stage motor symptoms of PD (Chagraoui et al., 2020). Still, it is also reported to cause high dyskinesia and relapse of PD symptoms (Turcano et al., 2018). Also, several disorders exhibit similar symptoms but vary in terms of mechanisms of their pathogenesis. These challenges demand an effective therapeutic intervention to slow down or halt the progression and present a permanent cure. Thus, researchers and clinicians have been looking for promising alternate, personalized, targeted therapeutic strategies to combat such neurodegenerative gait/postural impairments. This section highlights three current paradigms of neurodegenerative disease therapy design: Wearables, virtual reality (VR), and phytochemicals to improve neuro-motor functions.

### Wearable and Virtual Reality Technologies for Gait and Balance Rehabilitation

Taking a step forward from gait analysis, wearable devices are also being explored to assist/rehabilitate to tackle neurological gait disorders (Bowman et al., 2021). These devices perform real-time computation/detection based on kinematic, kinetic, and other physiological parameters and provide desired biofeedback (auditory, visual, vibratory, etc., cues) on detecting gait or postural anomaly (Mazilu et al., 2015; Lazarou et al., 2016; Pacilli et al., 2016; Pereira et al., 2016; Mendoza et al., 2017; Suppa et al., 2017; Amato et al., 2018; Rossi et al., 2020; Saif et al., 2020; Muurling et al., 2021; Stavropoulos et al., 2021). Such an approach influences motor learning in patients by engaging them toward continuous and real-time rehabilitation. Wearables, often compact and lightweight, possess a distinct advantage of being suitable for long-term and outdoor use, thereby enabling the user to experience and learn during realistic scenarios.

Virtual reality (VR) is another technology-driven paradigm shift in therapeutic intervention toward physical and cognitive rehabilitation of neurological disorders (Voinescu et al., 2021). VR-based rehabilitation technique creates a realistic experience by projecting virtual environments (through immersive/non-immersive displays). Physiotherapy is one of the earliest and most effective forms of therapy for gait and balance rehabilitation. However, it is a monotonous task that requires individuals to perform specific tasks repeatedly, and the outcome for such activity is not available for immediate realization. As a result, often, the individual loses interest in the activity. The VR-based intervention addresses this challenge by translating the standard therapeutic exercise protocols into interactive games (actions). The real-time measurement from the body segments (hands, leg, head, etc.) is used as biofeedback signals to update the therapy (game) environments. This results in better patient engagement in the physiotherapy session, ensuring a more effective outcome (Voinescu et al., 2021).

### Phytochemicals and Its Neuroprotective Roles

With the significant shortcomings associated with available drugs for the management of neurological disorders, researchers have been investigating the discovery of molecules that can effectively cure/prevent the pathology. The major pathological features associated with neuro-disorders are oxidative stress, neuro-inflammation, and aggregated proteins. Naturally, derived compounds from the plant (phytochemicals) have been widely used for extracting clinically valuable compounds. For example, the common flowering quince (FQ) has been used traditionally to treat migraine, neuralgia, depression, tremors, and dyskinesia (Zhao et al., 2008). Over the recent years, researchers have been exploring various phytochemicals like Berberine (BBR), Curcumin, Ginsenoside, Puerarin, etc., with potential application toward the management of neurodegenerative disorders and symptoms (Price et al., 2012; Lin et al., 2018; Corpas et al., 2019; Nair et al., 2019; Wang et al., 2019; Calfio et al., 2020; Balakrishnan et al., 2021). For example, phytochemical like Resveratrol (a bioactive component of red wine) (Price et al., 2012; Lin et al., 2018; Corpas et al., 2019; Nair et al., 2019) and Melatonin (Wang et al., 2019) have shown reported benefit, both *in vitro* and vivo conditions, toward improving the mitochondrial function believed to be one primary cause for diseases like AD and PD. Resveratrol, at a very low concentration, has also demonstrated its benefit toward preventing α-synuclein aggregation (Eschbach et al., 2015; Gautam et al., 2017). Apigenin (AGN), a flavone class phytochemical known for anti-inflammatory and free radical scavenging activities, is also reported to alleviate α-synuclein accumulation significantly and mitochondrial dysfunction in rotenone-induced PD rats (Anusha et al., 2017). Many studies reported promising results toward effective management of cognitive and non-motor functions (Balakrishnan et al., 2021). However, compounds such as BBR, quercetin, ferulic acid (FA), etc., have also shown promising motor functions in neuro-disorder induced rat and mice models (Chen et al., 2002; Van Kampen et al., 2003; Zhao et al., 2008; Kim et al., 2014; Jiang et al., 2015; Yadav et al., 2016; Askar et al., 2019; Madiha et al., 2021). For example, blueberry supplementation (rich in polyphenols) in the geriatric population effectively checks the decline of functional mobility and improves the performance of activities of daily living (Schrager et al., 2015). BBR showed effectiveness toward preventing memory loss in PD (Kim et al., 2014) and alleviating motor dysfunction in PD (Kim et al., 2014) and HD (Jiang et al., 2015) in mice models. Quercetin stands as a strong potential candidate for application in PD as it enhances antioxidant enzyme activity, thereby improving motor function (Madiha et al., 2021). When subjected to FA doses, rotenone-induced PD mice improved neuromotor function and muscle exercises (Askar et al., 2019). Curcumin, a polyphenol in turmeric, is one of the most promising natural compound against AD (Serafini et al., 2017; Reddy et al., 2018) owing to its β-amyloid inhibition and antioxidant solid property (Tang and Taghibiglou, 2017; Su et al., 2020; Utomo et al., 2021). An established ROS scavenger (Cao et al., 2008; Barzegar and Moosavi-Movahedi, 2011), curcumin also safeguards mitochondria against peroxynitrite in nigrostriatal, making it a strong therapeutic agent for AD and PD (Ramires Júnior et al., 2021). It is also reported to induce neuroplasticity in rats (Choi et al., 2017; Fan et al., 2018). Another widely extracted Phyto-compound, Ginseng, was reported to have improved dopaminergic neuronal loss and gait disturbance in PD mice models (Van Kampen et al., 2003). Table 3 summarizes the motor and biomechanical implications of common neurodegenerative gait disorders and the ongoing novel therapeutic approaches to mitigate the common gait and balance impairments accompanying them.

**TABLE 3:** Current progress in non-pharmacological therapeutic and rehabilitation measures for neurodegenerative gait and motor functions.

Neuro-disorder	Gait and biomechanical manifestation(s)	Advances in therapeutic/Caregiving strategies
AD	Slow gait speed Reduced step/stride length Low cadence Increased inter stride variability Bradykinesia	Wearables (Lazarou et al., 2016; Mendoza et al., 2017; Amato et al., 2018; Saif et al., 2020; Muurling et al., 2021; Stavropoulos et al., 2021), Virtual Reality (Gago et al., 2016; Doniger et al., 2018; Uğur and Sertel, 2020), Phytochemicals (Janßen et al., 2010)
PD	Freezing of gait Slow walking speed Small step length Bradykinesia Hypertonia (rigidity) Tremor Flexed posture Festination	Wearables (Mazilu et al., 2015; Pacilli et al., 2016; Pereira et al., 2016; Suppa et al., 2017; Rossi et al., 2020), Virtual Reality (Gilat et al., 2015; Killane et al., 2015; Georgiades et al., 2016; Bluett et al., 2019; Feng et al., 2019; Bekkers et al., 2020; Júlio et al., 2020; Pelosin et al., 2020; Yamagami et al., 2020; Goh et al., 2021), Phytochemicals (Van Kampen et al., 2003; Zhao et al., 2008; Kim et al., 2014; Yadav et al., 2016; Askar et al., 2019; Madiha et al., 2021)
HD	Slow gait speed Reduced stride length Variable stepping pattern Increased stance-to-swing ratio	Wearable (Kegelmeyer et al., 2017; Georgiou et al., 2020), Virtual Reality (Kloos et al., 2013; Júlio et al., 2020), Phytochemicals (Jiang et al., 2015)
ALS	Small stride length Decreased cadence Small single limb support Increased double limb support Increased knee flexion at IC Increased inter stride variability	Wearables (Garcia-Gancedo et al., 2019; Van Eijk et al., 2019), Virtual Reality (Ortiz et al., 2018), Phytochemicals (Mohi-Ud-Din et al., 2022)
MS	Decreased gait speed Small step length Reduced cadence Reduced joint ROM	Wearables (Peruzzi et al., 2017), Virtual Reality (Eftekharsadat et al., 2015; Kalron et al., 2016; Calabrò et al., 2017; Peruzzi et al., 2017; Munari et al., 2020)

## Discussion

Gait and balance analysis offer a medium to not only understand the locomotor/functional impairments but a way to accurately diagnose neurodegenerative disorders like PD, AD, MS, etc., that otherwise have almost similar non-motor symptoms. Despite being accurate and reliable, conventional gait laboratory-based measurement setups suffer from the limitation of being costly, bulky, and requires specialists to operate. Various research groups have researched WS-based gait analysis globally to address these challenges. We present a detailed review of all such recent progress (from 2005) in WS-based gait analysis based on the application of IMU, pressure sensor, EMG, FMG, Piezoelectric sensor, Microphone, and ultrasonic sensors along with available commercial systems. The review was performed covering certain clinically relevant aspects (parameters) of neurodegenerative gait disorders, and below, we summarize the role, applicability, and technique of the aforementioned sensors toward them. In addition, a summary of ongoing efforts toward mitigation of neurodegenerative gait disorders through application of wearable and VR technologies and the potential role of phytochemicals for alleviating such conditions have also been presented.

### Event Detection

Gait events like HS, TO, and FF are critical to gait partitioning and signify the phase shift between various walking states. Almost all reported sensors from IMUs to EMG are explored to detect such events in both healthy and impaired gait. Commonly used detection techniques include thresholding (in case of IMU, footswitches, pressure sensors, wearable microphone, and piezoelectric sensors), rate reversal/peak detection/zero-crossing (IMUs) (Jasiewicz et al., 2006; Raveendranathan et al., 2011; Yang et al., 2012; Hundza et al., 2013; Mariani et al., 2013; Joshi et al., 2016; Montero-Odasso and Perry, 2019), PSD and frequency analysis (for EMG and FMG) (Ryu and Kim, 2017; Ziegier et al., 2018; Morbidoni et al., 2019; Nazmi et al., 2019; Di Nardo et al., 2020; Keloth et al., 2021; Zahra, 2021; Rezaee et al., 2022). Researchers have focused on applying machine learning too for handling complex signals (from IMUs) associated with impaired gait for such event recognition.

### Kinematic Analysis

Joint and limb trajectories offer a proper way to understand the biomechanics of walking. Among all the reported sensors, IMU contributes to an overwhelming majority of research into understanding the kinematics of locomotion. From head to toe and range of motion to pose estimation, IMUs have been extensively used (Dorschky et al., 2019; Teufl et al., 2019a; Gholami et al., 2020; Yeo and Park, 2020). Recent effort toward the analysis of joint angles using instrumented force insole has also been reported using an artificial neural network (Li G. et al., 2016).

### Kinetic Analysis

Understanding of forces and torques associated with walking is equally important. The most common kinematic parameter explored in gait analysis is ground reaction forces. The force and pressure sensors embedded into footwear have been investigated by most researchers for healthy and impaired gait (Marcante et al., 2021; Pardoel et al., 2021; Shalin et al., 2021). Recently, the application of inertial sensors has also been studied for the estimation of GRF and joint moments (Kodama and Watanabe, 2016). EMG and FMG are the other two techniques commonly used for measuring muscle forces.

### Spatiotemporal Analysis

Gait presents a series of time and length domain data crucial for quantitative and qualitative gait evaluation. Although almost all the mentioned sensors capable of event detection are useful for temporal parameter measurement, IMUs integrated with a human model are reported widely toward estimation of spatial parameters such as step/stride length and step width (Salarian et al., 2012; Takeda et al., 2014; Ferrari et al., 2015; Rampp et al., 2015; Wang and Ji, 2015; Liu et al., 2016; Hao et al., 2019; O’brien et al., 2019; Teufl et al., 2019a; Yeo and Park, 2020; Das and Kumar, 2021).

### Balance Analysis

Center of mass, CoG, and postural sway are the most common indicator of postural balance corresponding to gait and stance. Instrumented foot insoles are the most commonly used technique for measuring (Marcante et al., 2021; Pardoel et al., 2021; Shalin et al., 2021), along with a few reports using IMUs (Hsu et al., 2014; Noamani et al., 2020; Dugan et al., 2021). In addition, such methods are also reportedly used for automated and quantified measures of standard balance scales such as 10 MWT, BBS, 2 min standing test, and TUGT (Wang Z. X. et al., 2015; Kodama and Watanabe, 2016; Noamani et al., 2020; Dugan et al., 2021). However, gait and balance analysis have already been commonly adopted for physiological and functional monitoring; limited reliable and straightforward wearable devices are used in non-specialized clinical settings. Realistic implementation of WS in the clinical setting demands limiting the number of sensor devices for long-term monitoring. Although a more significant number of sensors placed at different body segments yields higher spatial features, at the same time, it increases computational as well as data analysis complexity along with system cost. Moreover, the outcome/output from a gait analysis system should contain clinically meaningful and comprehensive gait features. To minimize clinicians’ temporal, physical, and cognitive burdens, it is highly desirable to have the fewest number of devices possible to assess performance. Although the state-of-art commercially available systems provide widely accepted gait parameters, they lack objectivity in data interpretation. Clinicians analyze the data based on their expertise and experience to draw inferences for disorder correlation. This is a time-consuming and burdensome process and often suffers from subjectivity limitation. An added “intelligence” to the WS-based measurement to automatically correlate normal and pathological gait would assist the clinicians in better evaluation of the derived gait features. Progress has been made in research in automatic neurodegenerative disorder classification using gait features during the last few years. However, most reported research on gait impairment identification algorithms involves input features from laboratory-based measurement (NWS) systems.

### Therapeutic Advances

Although the exact pathogenesis of most neurodegenerative disorders like PD, AD, MS, ALS, etc., couldn’t be ascertained yet, some distinctive hallmarks have been associated with them. The common pathologies reported includes mitochondrial dysfunction (common in AD, PD, and MS) (Correia et al., 2012; Carvalho et al., 2015; Rahman and Rhim, 2017), accumulation of misfolded α-synuclein (Chen et al., 2019), aggregation of Amyloid-β (Aβ) senile plaques (Malafaia et al., 2021), up of dopamine (DA) depletion (Bastide et al., 2015), neuroinflammation, oxidative stress, and induced endoplasmic reticulum (ER) stress, formation of reactive oxygen species (ROS) (Manoharan et al., 2016), to autoimmune-mediated loss of myelin and axonal damage (Jones et al., 2017), etc. Numerous bioactive phytochemicals have gained special attention as potential neuroprotective agents that help in mitigating such associated pathologies. For example, Curcumin (present in turmeric) exhibits multitude of benefits including antioxidant, β-amyloid inhibition property (Tang and Taghibiglou, 2017; Su et al., 2020; Utomo et al., 2021), ROS scavenging (Cao et al., 2008; Barzegar and Moosavi-Movahedi, 2011), alleviating mitochondrial damage (Ramires Júnior et al., 2021). Similarly, AGN has shown usefulness through anti-inflammatory and free radical scavenging activities, which in turn clear α-synuclein accumulation (Anusha et al., 2017). Mitochondrial function improvement has been reported using Resveratrol (Price et al., 2012; Lin et al., 2018; Corpas et al., 2019; Nair et al., 2019), AGN (Wiik, 2016), Curcumin (Ramires Júnior et al., 2021), Apigenin (Anusha et al., 2017), Curcumin (Cao et al., 2008; Barzegar and Moosavi-Movahedi, 2011), Quercetin (Madiha et al., 2021) are the most commonly investigated compounds showing strong anti-oxidant properties.

Other than pharmacological therapeutic interventions, efforts are also underway to provide a solution to tackle some of the significant motor challenges associated with neuro-disorders through VR and wearable devices. Both this class of devices performs real-time analysis of body movements (including gait) and generates various forms of countermeasure signals. The major benefit such approach offers is it provides a way for motor and cognitive learning, thereby ensuring effective and long-term rehabilitation.

## Future Direction

For over two decades, tremendous efforts in wearable gait analysis have shown some fruitful results and translation to commercial products like XSENS F-Scan. Currently, such systems are parallelly used along with the gold standards like force and motion capture systems. However, these systems’ broad and consensual clinical applicability demands further work and improvements. As a takeaway from the literature review performed, below are some of the critical suggestions presented by the authors toward effective, easy-to-use WS devices for regular use in clinical practice.

Firstly, the non-uniformity of WS-based measurement methods and outcome measures needs to be addressed. There is no consensus concerning the number of sensors, position, placement on the body, resultant gait feature set, etc. Such an approach hinders scaling up for general and broad applicability. Measurement devices need to generate reliable and reproducible output for clinical and research use.

Secondly, there is a need to develop intelligent and intelligent algorithms, especially concerning the umbrella of disorders diagnosis. Most reported works (algorithms) exploit certain traits associated with a particular disease, making them disease-specific and less efficient with other disorders. However, the system needs to cater to the broader population group for clinical applicability. Moreover, most of the reported literature deals with binary (sometimes 3–4) classification, i.e., targeted to identifying a selected disorder. Classification of conditions based on the severity, i.e., grading the disease, is still an area that needs broader investigation for acceptance in clinical usability. An objective, automated process of quantifying and classifying gait data can allow clinicians to invest their resources and time in prescribing better, more effective, and more targeted treatments in actual clinical practice. More penetration of AI, ML, and deep learning techniques can bridge the gap.

And thirdly, the exploitation of smartphones as a tool for movement analysis requires more focus. Given the broad reach of smartphone devices for extensive use, such mobile-based technologies can cater to a large population with limited access to quantified gait analysis. Moreover, accurate life data from all daily activities can open up newer avenues in this direction.

## Conclusion

This article highlights significant recent works toward assessing neurodegenerative gait disorders using wearable sensing techniques. Some recent progress reported toward non-pharmacological therapeutic intervention toward mitigating gait and balance disorders originating from neuro-degeneration. Reported sensing methods for studying gait kinematics, kinetics, spatiotemporal, and postural balance through inertial, footswitches, pressure sensors, ultrasound sensors, proximity sensors, plantar pressure sensors, electromyography, etc., have extensively been discussed. The IS-based measurements correlate well with kinematics and other qualitative and quantitative measures corresponding to gait and activity monitoring. IMUs, owing to distinct advantages, offer the most promising alternative to laboratory-based gait analysis covering almost all spectrum of clinical gait analysis. The wide availability of IMU integrated devices is undoubtedly a positive aspect of translating such technologies for clinical benefit. However, specific challenges still need to be addressed toward achieving that goal, mainly deriving a common technology consensus and guideline conforming to its use. The wearable biofeedback systems and VR-based technology offer a promising solution toward rehabilitation and assistance of neuro-impaired gait symptoms, such as in the case of freezing of gait episodes postural imbalance. Similarly, various phytochemical compounds such as BBR FA have shown few positive outcomes in mitigating movement and balance-related impairments in cellular and rodent models. However, research in this domain is still pre-infancy and demands a widescale effort to reach a firm conclusion concerning their benefit in tackling neurodegenerative gait disorders in humans.

## Author Contributions

RD performed the literature search article scrutiny and wrote the first draft of the manuscript. All authors contributed to the conception and design of the study, and reviewed, read, and approved the submitted version.

## Conflict of Interest

The authors declare that the research was conducted in the absence of any commercial or financial relationships that could be construed as a potential conflict of interest.

## Publisher’s Note

All claims expressed in this article are solely those of the authors and do not necessarily represent those of their affiliated organizations, or those of the publisher, the editors and the reviewers. Any product that may be evaluated in this article, or claim that may be made by its manufacturer, is not guaranteed or endorsed by the publisher.

## Abbreviations

10 MWT: 10 meter walk test
2D: two dimensional
3D: three dimensional
AA: ankle arthrodesis
AD: Alzheimer’s disease
ALS: amyotrophic lateral sclerosis
AO: ankle orthosis
BBS: Bergs Balance Scale
BF: biceps femoris
CF: complementary Filter
CMT: Charcot-Marie-Tooth
CNN: convolution neural network
CoG: center of gravity
CoM: center of mass
CoP: center of pressure
EMG: electromyography
ES: erector spinae
FF: flat foot
FFT: Fast Fourier transform
FMG: force myography
FSR: force sensitive resistors
GCT: gait cycle time
GM: gastrocnemius medialis
GRF: ground reaction force
HC: healthy controls
HD: Huntington’s disease
HMM: Hidden Markov Model
HO: heel off
HS: heel strike
IC: initial contact
IMU: Inertial Measurement Unit
IPM: inverse pendulum model
IS: inertial sensors
KF: Kalman Filter
LDA: linear discriminant analysis
L_*stp*_: step length
MEMS: micro-electromechanical systems
MS: multiple sclerosis
ms: millisecond
MSt: mid-stance
NWS: non-wearable sensors
OMC: optical motion camera
PD: Parkinson’s disease
PSw: pre-swing
PVDF: polyvinylidene fluoride
RF: rectus femoris
ROM: range of motion
SCI: spinal cord injury
sEMG: surface-electromyography
SL: stride length
STP: spatiotemporal parameter
St_*t*_: stance time
SV: stride velocity
SVM: support vector machine
Sw: swing
TA: tibialis anterior
TAR: total ankle replacement
TO: toe off
TS: toe strike
T_*Sw*_: swing time
TUGT: time-up and go test
vGRF: vertical ground reaction force
VM: vastus medialis
WFSM: wireless foot sensor module
WHO: World Health Organization
WS: wearable sensors
ZUPT: zero update
μ V: micro volts.
